# Preoperative Anxiety in the Surgical Transfer and Waiting Area: A Cross-Sectional Mixed Method Study

**DOI:** 10.3390/jcm11092668

**Published:** 2022-05-09

**Authors:** Mikhail Dziadzko, Tessa Mazard, Myriam Bonhomme, Mahé Raffin, Pierre Pradat, Jean-Marc Forcione, Raphael Minjard, Frederic Aubrun

**Affiliations:** 1 Département d’Anesthésie-Réanimation, Hôpital de la Croix-Rousse, Hospices Civils de Lyon, Université Claude Bernard, 69004 Lyon, France; tessa.mazard@chu-lyon.fr (T.M.); myriam.bonhomme@outlook.fr (M.B.); jean-marc.forcione@chu-lyon.fr (J.-M.F.); frederic.aubrun@chu-lyon.fr (F.A.); 2 INSERM U1290 RESHAPE, Université Claude Bernard Lyon 1, 69622 Lyon, France; 3 Consultation Douleur, Groupement Hospitalier Nord, Hospices Civils de Lyon, 69005 Lyon, France; r_minjard@msn.com; 4 Centre de Recherche Clinique, Hôpital de la Croix-Rousse, Hospices Civils de Lyon, Université Claude Bernard, 69004 Lyon, France; mahe.raffin@chu-lyon.fr (M.R.); pierre.pradat@chu-lyon.fr (P.P.); 5 Centre de Recherche en Psychopathologie et Psychologie Clinique, Université Lumière Lyon-II, 69500 Bron, France

**Keywords:** holding area, nurse intervention, preoperative anxiety, psychical envelope

## Abstract

Severe preoperative anxiety (SPA) in surgical patients may have clinical consequences and worsen satisfaction. Some institutions have a surgical transfer and waiting area (TWA) for patient reception/dispatch to operating rooms. We measured the frequency of SPA, investigated predicting factors, and quantified the effect of the TWA stay on anxiety levels in a single centre cross-sectional study. Preoperative anxiety was assessed using three scales. Patients’ perceptions/suggestions were studied by a psychoanalyst. A total of 933 adult patients, undergoing elective procedures, were interviewed. SPA was detected in 24.7%, non-modified by anxiolytic premedication. Patients’ median stay was 9 min, and anxiety level was decreasing in those with SPA. In multivariable analysis, female sex, inpatient settings, and pain before the procedure were predictive for SPA. Previous operating room experience, and a supine arrival position were associated with less SPA. Patients complained about a lack of information, and an uncomfortable environment in the waiting area. To reduce anxiety, they mainly asked for warm blankets/music (physical/sound barriers), and extra sedative agents. The holding area may be a place to measure patients’ anxiety by paramedical staff, and to apply simple non-pharmacological interventions. The psychological concept of psychical envelopes may be useful for the development and investigation of such interventions in improving patients’ experience.

## 1. Introduction

Previous studies have demonstrated a high prevalence (25–45%) of severe preoperative anxiety (SPA) in the general population [1], leading to acute distress, increased anaesthetic requirements, delayed recovery, increased post-procedural pain, nausea and vomiting, psychological distress, and non-satisfaction [2,3].

Although the phenomenon of preoperative anxiety is universal, several non-modifiable (sex, age, medical history, scheduled surgery) and modifiable predictive factors (pain, negative future perception, high trait-anxiety) were identified [1,4]. It has been shown that systematic anxiolysis modestly improves the patient experience [5]. However, selective anxiolytic administration based on preoperative anxiety evaluation may be considered [6,7].

Some institutions have a surgical transfer and waiting area (TWA) for patient reception and dispatch to operating rooms (OR). This busy environment can induce uncertainty and additional discomfort for a patient; however, it can also be a place for patients to improve their experience.

Few studies address the acute preoperative anxiety in the surgical holding area. Most of them focus on prevalence, time-trend and predictive factors, which are either not modifiable or difficult to amend during the time of the patient’s stay in the TWA [8].

We conducted a cross-sectional study to evaluate the frequency of SPA and to determine how the anxiety level changes during a patient’s stay in the holding area. Additionally, we recorded and analysed patient experiences at the TWA from psychoanalytical perspectives.

## 2. Materials and Methods

**Figure A1:**
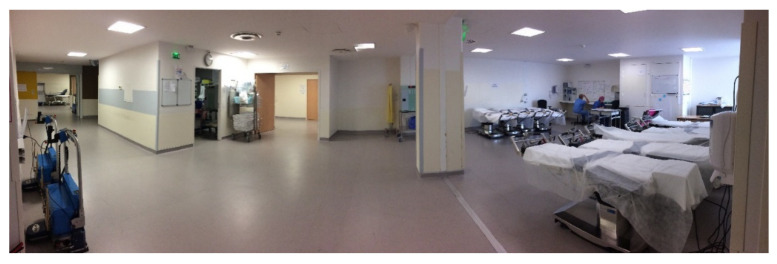
Picture of a surgical transfer and waiting area. Patients arrive from the floor (left), received by a paramedical staff and transferred to the surgical tables (right, far corner), and are then dispatched to the ORs (exit, center). The PACU entrance/exist is just behind the central spot.

A cross-sectional, single-centre cohort study was conducted in a tertiary academic centre, Hôpital de la Croix Rousse, Hospices Civils de Lyon. Twenty operating rooms, geographically located on the same floor, have a common TWA. This area represents a hallway with the entrance and two exits toward the operating rooms and post anaesthesia care unit—Figure A1 Appendix A. Patients arrive in a supine position (bed or stretchers), in wheelchairs or walking from their hospitalization locations/wards. They are then installed on surgical tables and dispatched to their destination OR (gastrointestinal, orthopaedics, ear and nose, eye, and plastic surgery). The surgical TWA has a 10-bed capacity, and is managed by a paramedical staff.

Consecutive patients passing through the TWA were invited to participate on their arrival to the TWA. Inclusion criteria were adult age (≥18 years old), elective surgery or procedure, agreeing to participate and ability to consent.

Those who agreed were questioned during their stay at the TWA. Interrogations included the measurement of anxiety using three scales (the Amsterdam preoperative anxiety and information scale (APAIS) [9], Covi’s anxiety scale (COVI) [10], and the visual analogue scale for anxiety (VAS-A) [6]), and patients’ perceptions/suggestions regarding their transition through the TWA. Exclusion criteria were minor patients, emergency surgery or procedure, refusal to participate, and failure to obtain consent.

The primary outcome was defined as the proportion of patients with SPA. The preoperative level of anxiety was assessed using the French version of the six-item APAIS [9,11] upon arrival. APAIS is a patient-reported outcome measurement, containing two domains (anaesthesia and surgery) and exploring three aspects of preoperative anxiety (worrying, thinking, and the need of information). Questions are rated using a Likert scale from 1 (least) to 5 (worst), resulting in a score ranging from 6 to 30. The cut-off for SPA was defined as APAIS ≥ 11 [9].

Secondary outcome measurements included the measurement of anxiety using Covi’s anxiety (COVI) scale. The COVI scale is a hetero-evaluation tool, contains three parts that assess the severity of anxiety in terms of the patient’s verbal report, behaviour, and somatic symptoms ranging from 1 (not at all) through to 5 (very much) [10].

Other measurements included the length of TWA stay, changes in preoperative anxiety, patients’ modes of arrival to the TWA, satisfaction with the arrival mode, current pain level, perceptions of care, and suggestions to reduce their preoperative anxiety.

To measure a potential effect of the TWA stay on patient anxiety we used an 11-level VAS-A on arrival and before departure from the TWA to the OR. Such a scale has a good correlation with commonly used anxiety measurement scales [6,12]. A two-point changes in VAS-A measurement was considered to be clinically significant [13,14]. All mentioned data were collected prospectively at the bedside of patients in the TWA by trained nurses using digital tablets and an electronic case report form (Ennov Clinical, Ennov Paris, France).

Demographic (age, sex, body mass index) and medical data (ASA status, out/inpatient status, hospitalization >24 h, previous OR experience, type of scheduled procedure, expected perioperative consciousness, received anxiolytic premedication, and the time spent in the TWA, were collected from the institutional electronic health record at the bedside.

Surgical and diagnostic procedures were classified by the anticipated pain level (no or weak pain: cataract surgery, gastroscopy; moderate to severe pain: meniscal resection, laparoscopic cholecystectomy, shoulder arthroplasty, pancreatic surgery etc.) [15]. There were two levels for the anticipated perioperative consciousness: “no” for general anaesthesia or sedation, and “yes” for local/regional anaesthesia alone. Open questions regarding patients’ perception of care were transcribed and pile sorted, and were then analysed by a psychoanalyst.

A comparison between patients with severe and non-severe anxiety was performed using the Wilcoxon rank sum test or Fisher’s exact test, as appropriate. A logistic regression analysis was performed to explore the potential factors associated with SPA as a planned post hoc analysis. A minimal sample size of 369 patients was estimated, assuming the prevalence of SPA as 30%, with a 95% confidence interval and 5% margin of error. As we planned to explore potential associations of SPA factors, we increased the sample size up to 950 patients using the rule of events per variable [16]. A two-tailed *p*-value < 0.05 was used to define statistical significance. Statistical analyses were performed using JMP 11 (SAS, Cary, NC, USA) software.

According to French legislation effective at the time of this observational study, the opinion of an ethics committee was not mandatory; patient oral informed consent and non-opposition were sufficient for inclusion. This study has been registered as clinical trial NCT02811107.

## 3. Results

**Figure 1:**
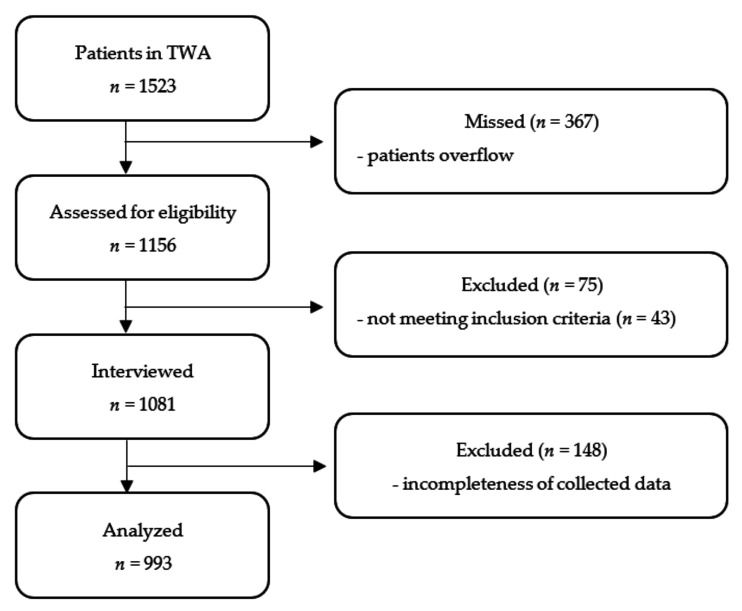
The study flowchart. TWA, transfer and waiting area.

Patient enrolment started in March 2016, and was completed in April 2016. During the study, 1523 patients were admitted to the surgical TWA, of whom, 367 patients were missed because of overflow. Of the 1156 patients who were approached, 1081 were interviewed (75 were excluded because they did not meet the inclusion criteria (*n =* 43) or declined to participate (*n =* 32)). After the whole data extraction, 148 patients had to be excluded because of the incompleteness of collected data (a technical issue with the electronic case report form), leaving 933 patients for the final analysis (Figure 1).

SPA defined with APAIS ≥ 11 was detected in 230 (24.7%) patients. There were strong positive correlations between all three scales used to measure patients’ anxiety levels (Table 1).

Patients with SPA were younger, more frequently female, had fewer comorbidities (ASA I), had more frequently experienced an OR environment, were more frequently in pain before a procedure, and were more frequently scheduled for procedures with anticipated moderate to severe postoperative pain. Patients admitted on a stretcher had lower SPA (Table 2).

Anxiolytic agents (short acting benzodiazepines and/or hydroxyzine) did not affect the frequency of SPA. There was no significant difference in the proportion of patients with SPA according to whether they were scheduled for a conscious procedure (local/regional anaesthesia) or unconscious procedure (sedation/general anaesthesia).

In the multivariable analysis, female sex, inpatient settings, and the presence of pain before the procedure were risk factors for SPA; admission in a supine position (especially on stretchers) was associated with lower prevalence of SPA (Table 2).

Patients’ median [IQR] length of stay in the TWA was 9 [5 to 16] min. Upon arrival, the median [interquartile range, IQR] APAIS score was 7 [5 to 10] and VAS-A 3 [1 to 5]; the correlation coefficient (rho) between these scores was 0.65 (*p* < 0.0001). The median VAS-A score on departure from the TWA was 3 [IQR 1 to 5], not significantly different from the VAS-A on arrival (*p* = 0.3186). Clinically relevant anxiety level changes during a patients stay in the holding area were detected in 223 (24%) patients, mostly in those with an APAIS score ≥ 11 at arrival (76 (33%), *p* = 0.0003). Results are summarized in Table 3.

The duration of the TWA stay did not predict significant changes in anxiety measured with VAS (*p* = 0.5891).

Regarding patients’ preferences to be admitted to the TWA, 75.7% preferred arrival directly in a bed, and only 32.4% preferred a walking arrival. Warm blankets (65.9%), music (58.2%), and extra sedatives (44.3%) were the factors most frequently suggested to reduce anxiety; few patients suggested screens (18%) or reading (18%). Two hundred and ten (22.5%) expressed their perception of the surgical TWA experience. Most of them (177 patients, 84.2%) complained of discomfort associated with cold/noisy environment, long waiting time, uncomfortable installation, lack of privacy, or disruptive smells. Thirty-three patients were not confident because of the busy environment (22 patients), or because of a lack of information or appropriate relations with clinicians (11 patients).

## 4. Discussion

The present study found, in a heterogeneous group of patients, that a quarter of those transiting through the TWA experienced SPA. The TWA stay resulted in clinically relevant anxiety changes, especially in patients with SPA. In the multivariable analysis, SPA on arrival was associated with female sex, the presence of pain before the procedure, and the inpatient setting; it was not associated with anxiolytic premedication.

These findings concur with the published studies [1,4,5,17]. Surgical patients’ anxiety in the transfer and waiting (or holding) area has already been studied by several teams. However, the reported prevalence in SPA varies because of different measurement tools and cut-offs used [8,18,19,20]. Although the APAIS score addresses the common dimensions of preoperative anxiety (fear of anaesthesia and procedure, with the need of information), the significance of the SPA cut-off may be different regarding patients groups and countries [21]. Regardless the absolute value of the threshold for SPA and the instrument of measurement, it allows identification of anxious patients in the TWA who may need extra support.

In our study, 20% of patients with SPA have shown a clinically significant decrease in their anxiety level over the time of transition through the holding area. This could be explained by an interactive effect of the study—patients were given attention from interviewing staff. This suggests that patients may benefit from express anxiety level testing on arrival, and their experience could be improved by cognitive interventions or alternative medicine shortly before the interventions [18].

The presence of pain (measured as >3 out of 10) in the TWA was associated with SPA, which is consistent with previous findings. Routine “protective premedication” combining benzodiazepines and opioids is no longer recommended in the current anaesthesia practice. Patients experiencing acute or chronic pain have mood dysregulation and a higher anxiety level [22], which is, in turn, associated with an increased awareness of surrounding threats and perceived pain [23]. As such, conditional non-opioid analgesic premedication may be considered in patients experiencing pain who are scheduled for surgery.

Surprisingly, patients’ admission to the TWA in a supine position (stretcher or bed) was associated with lower prevalence of SPA. Enhanced patient autonomy by means of outpatient settings [24] and walking to the operating room [25,26] has been shown to reduce preoperative anxiety and improve patient satisfaction. At the time the study was conducted, patients were not systematically informed about their OR admission mode, which may explain this disagreement with published results.

Several non-pharmacological methods have been explored to reduce SPA, for instance music, preoperative warming, video distraction, and aromatherapy [27]. In the present study, patients suggested blankets and music as means to reduce their anxiety. From the psychoanalytical view, such requests can be explained using the *envelope concept* [28]. The enveloping induced by a warm blanket allows the patient to feel the limits of their body and to contain its anxieties. The music creates a reassuring sound envelope in front of the sounds of the hospital, experienced as cold, deadly and intrusive. The capacity model of psychoanalytical care explains the creation of a dedicated reservoir to contain or to confine unpleasant or painful emotions [29]. The human encounter makes a fundamental difference between “blankets or music available” and “offering a blanket or music to someone” by promoting an exchange of words and touch [30]. For this reason, blankets or music may be considered as tools for caregivers to enhance the patient experience.

Some limitations of the study need to be acknowledged. Different potential confounders were not explored, including patients’ education and income levels, as well as their history of mood/psychiatry disorders, which may have affected the occurrence of SPA in this population. Anxiolytic premedication was prescribed during pre-anaesthesiology evaluation more than 48 h prior to the intervention, at the anaesthesiologists’ discretion, without a systematic evaluation of anxiety, therefore we were unable to analyse the relevance of prescribed drugs. The type of anxiolytic premedication, as well as the interval between premedication administration and arrival at the TWA, were not included in the analysis. The study involved only adult patients scheduled for elective procedures; however, the nature of preoperative anxiety in children and in the case of emergency surgery is probably more complex to analyse. No intervention was tested in our study, but we observed a positive effect from a simple interaction with patients. Cross-sectional design of the study predisposes to bias occurrence, such as selection/ascertainment and information bias. The study used non-probability sampling, however, we included consecutive patients with a large sample size. Standardised and validated methods of anxiety measurement were used (validated scales). Confounding may be an important issue. We used a multiple logistic regression to control this issue.

## 5. Conclusions

In conclusion, a quarter of patients suffer from SPA in the TWA. SPA can be identified using simple tools. In patients with preprocedural pain, SPA was detected more frequently, suggesting the need of analgesic premedication. About 20% of patients have clinically relevant changes in their anxiety level during their stay in the holding area. The duration of the patient’s stay in the TWA may be sufficient to apply simple interventions to reduce SPA. The concept of psychical envelopes may be used for the development and investigation of such interventions, to improve patient experiences and to reduce harm from SPA.

## Figures and Tables

**Table 1:** Results of anxiety level measurements in patients upon TWA arrival and correlation between measurement instruments.

	APAIS 7 [5 to 10]	VAS-A 3 [1 to 5]	COVI 3 [2 to 5]
APAIS	1	ρ 0.65, *p* < 0.0001	ρ 0.58, *p* < 0.0001
VAS-A		1	ρ 0.54, *p* < 0.0001
COVI			1

Measurement values for anxiety are presented as median and [interquartile range]. TWA, transfer and waiting area; APAIS, Amsterdam preoperative anxiety and information scale; COVI, Covi’s anxiety scale. Data shown as medians [interquartile range], and Spearman’s Rho.

**Table 2:** Factors potentially associated with severe preoperative anxiety in the transfer and waiting area (*n =* 933).

		Univariable Analysis			Multivariable Analysis	
	All Patients *n* (% of Total)	APAIS ≥ 11 *n =* 230 (24.7%)	APAIS < 11 *n =* 703 (75.3%)	*p*-Value	Odds Ratio (95%CI)	*p*-Value
Age, years	61 [45 to 72]	58 [41 to 70]	62 [47 to 73]	0.0107	0.99 (0.98 to 1.01)	0.4263
Sex [F]	435 (46.6%)	141 (32.4%)	294 (67.6%)	<.0001	2.29 (1.63 to 3.21)	<0.001
BMI, kg/m^2^	24.6 [21.6 to 27.7]	24.22 [21.5 to 27.4]	24.8 [21.6 to 27.9]	0.3425	0.99 (0.96 to 1.02)	0.5819
ASA class I vs. ≥ II	280 (30%)	87 (31.1%)	193 (68.9%)	0.0037	1.44 (0.93 to 2.23)	0.0978
Inpatient setting (Y)	588 (63.0%)	157 (26.7%)	431 (73.3%)	0.0594	2.13 (1.34 to 3.39)	0.0014
Hospitalisation > 24 h(Y)	123 (13.2%)	23 (18.7%)	100 (81.3%)	0.1158	0.71 (0.40 to 1.25)	0.2357
Previous OR experience (Y)	868 (93.1%)	206 (23.7%)	662 (76.7%)	0.0348	0.56 (0.29 to 1.05)	0.0698
Anxiolytic premedication (Y)	114 (12.2%)	34 (29.8%)	80 (70.2%)	0.2015	1.19 (0.73 to 1.96)	0.4772
Pain (NS > 3) in the TWA (Y)	160 (17.2%)	50 (31.3%)	110 (68.7%)	0.0434	1.57 (1.04 to 2.37)	0.0336
Procedures with preserved consciousness (Y)	473 (50.7%)	110 (23.3%)	363 (76.7%)	0.3242	0.80 (0.56 to 1.13)	0.2056
Anticipated pain level associated with procedure						
No pain to light pain vs. moderate to severe pain	413 (44.3%)	81 (19.6%)	332 (80.4%)	0.0017	1.33 (0.87 to 2.03)	0.1839
Arrival mode						
Walking	71 (7.6%)	17 (23.9%)	54 (76.1%)	1	1 (1 to 1)	1
Wheelchair	63 (6.8%)	18 (28.6%)	45 (71.4%)	0.4512	0.97 (0.36 to 2.63)	0.9453
Stretcher	120 (12.8%)	18 (15%)	102 (85%)	0.0088	0.29 (0.11 to 0.75)	0.0110
Bed	679 (72.8%)	177 (26.1%)	502 (73.9%)	0.1055	0.42 (0.18 to 1.02)	0.0543
Supine Position	799 (85.6%)	195 (24.4%)	604 (75.6%)	0.6658	0.36 (0.18 to 0.72)	0.0035
Satisfaction with the arrival mode (Y)	775 (83.1%)	200 (25.8%)	575 (74.2%)	0.0847	1.14 (0.60 to 1.87)	0.5977

Continuous variables are presented as medians and interquartile ranges [IQR]. Categorical variables are presented as counts and percentages. F, female; ASA, American Society of Anaesthesiology; Y, yes; OR, operating room; NS, numeric scale; TWA, transfer and waiting area.

**Table 3:** Clinically relevant changes in anxiety level (>2 points) in patients during their stay in the TWA.

VAS-A Changes	All Patients *n =* 933	APAIS ≥ 11 *n =* 230	APAIS ≤ 11 *n =* 703	
Increase	96 (10.29%)	27 (11.7%) χ^2^ = 0.47	69 (9.8%) χ^2^ = 0.15	*p* = 0.0003 *
Decrease	127 (13.61%)	49 (21.3%) χ^2^ = 1	78 (11.1%) χ^2^ = 3.27	
No changes	710 (76.1%)	154 (67%) χ^2^ = 2.52	556 (79.1%) χ^2^ = 0.83	

VAS-A, visual analogue scale for anxiety; APAIS, Amsterdam preoperative anxiety and information scale; * significant difference across the table, Pearson’s chi-squared test.

## Data Availability

All data generated in this study are property of the Hospices Civils de Lyon and not will be shared.

## References

[1] Aust H., Eberhart L., Sturm T., Schuster M., Nestoriuc Y., Brehm F., Rusch D. (2018). A cross-sectional study on preoperative anxiety in adults. J. Psychosom. Res..

[2] Kain Z.N., Sevarino F., Alexander G.M., Pincus S., Mayes L.C. (2000). Preoperative anxiety and postoperative pain in women undergoing hysterectomy. A repeated-measures design. J. Psychosom. Res..

[3] Kil H.K., Kim W.O., Chung W.Y., Kim G.H., Seo H., Hong J.Y. (2012). Preoperative anxiety and pain sensitivity are independent predictors of propofol and sevoflurane requirements in general anaesthesia. Br. J. Anaesth..

[4] Eberhart L., Aust H., Schuster M., Sturm T., Gehling M., Euteneuer F., Rusch D. (2020). Preoperative anxiety in adults—A cross-sectional study on specific fears and risk factors. BMC Psychiatry.

[5] Maurice-Szamburski A., Auquier P., Viarre-Oreal V., Cuvillon P., Carles M., Ripart J., Honore S., Triglia T., Loundou A., Leone M. (2015). Effect of sedative premedication on patient experience after general anesthesia: A randomized clinical trial. JAMA.

[6] Kindler C.H., Harms C., Amsler F., Ihde-Scholl T., Scheidegger D. (2000). The visual analog scale allows effective measurement of preoperative anxiety and detection of patients’ anesthetic concerns. Anesth. Analg..

[7] Miller K.M., Wysocki T., Cassady J.F., Cancel D., Izenberg N. (1999). Validation of measures of parents’ preoperative anxiety and anesthesia knowledge. Anesth. Analg..

[8] Kumar A., Dubey P.K., Ranjan A. (2019). Assessment of Anxiety in Surgical Patients: An Observational Study. Anesth. Essays Res..

[9] Moerman N., van Dam F.S., Muller M.J., Oosting H. (1996). The Amsterdam Preoperative Anxiety and Information Scale (APAIS). Anesth. Analg..

[10] Lipman R.S. (1982). Differentiating anxiety and depression in anxiety disorders: Use of rating scales. Psychopharmacol. Bull..

[11] Maurice-Szamburski A., Loundou A., Capdevila X., Bruder N., Auquier P. (2013). Validation of the French version of the Amsterdam preoperative anxiety and information scale (APAIS). Health Qual. Life Outcomes.

[12] Boker A., Brownell L., Donen N. (2002). The Amsterdam preoperative anxiety and information scale provides a simple and reliable measure of preoperative anxiety. Can. J. Anaesth..

[13] Farrar J.T., Portenoy R.K., Berlin J.A., Kinman J.L., Strom B.L. (2000). Defining the clinically important difference in pain outcome measures. Pain.

[14] Mouelhi Y., Jouve E., Castelli C., Gentile S. (2020). How is the minimal clinically important difference established in health-related quality of life instruments? Review of anchors and methods. Health Qual. Life Outcomes.

[15] Gerbershagen H.J., Aduckathil S., van Wijck A.J., Peelen L.M., Kalkman C.J., Meissner W. (2013). Pain intensity on the first day after surgery: A prospective cohort study comparing 179 surgical procedures. Anesthesiology.

[16] Peduzzi P., Concato J., Kemper E., Holford T.R., Feinstein A.R. (1996). A simulation study of the number of events per variable in logistic regression analysis. J. Clin. Epidemiol..

[17] Mavridou P., Dimitriou V., Manataki A., Arnaoutoglou E., Papadopoulos G. (2013). Patient’s anxiety and fear of anesthesia: Effect of gender, age, education, and previous experience of anesthesia. A survey of 400 patients. J. Anesth..

[18] Attias S., Keinan Boker L., Arnon Z., Ben-Arye E., Bar’am A., Sroka G., Matter I., Somri M., Schiff E. (2016). Effectiveness of integrating individualized and generic complementary medicine treatments with standard care versus standard care alone for reducing preoperative anxiety. J. Clin. Anesth..

[19] Matthias A.T., Samarasekera D.N. (2012). Preoperative anxiety in surgical patients—Experience of a single unit. Acta Anaesthesiol. Taiwanica.

[20] Pokharel K., Bhattarai B., Tripathi M., Khatiwada S., Subedi A. (2011). Nepalese patients’ anxiety and concerns before surgery. J. Clin. Anesth..

[21] Buonanno P., Laiola A., Palumbo C., Spinelli G., Terminiello V., Servillo G. (2017). Italian validation of the Amsterdam Preoperative Anxiety and Information Scale. Minerva Anestesiol..

[22] Michaelides A., Zis P. (2019). Depression, anxiety and acute pain: Links and management challenges. Postgrad. Med..

[23] James J.E., Hardardottir D. (2002). Influence of attention focus and trait anxiety on tolerance of acute pain. Br. J. Health Psychol..

[24] Wetsch W.A., Pircher I., Lederer W., Kinzl J.F., Traweger C., Heinz-Erian P., Benzer A. (2009). Preoperative stress and anxiety in day-care patients and inpatients undergoing fast-track surgery. Br. J. Anaesth..

[25] Humphrey J.A., Johnson S.L., Patel S., Malik M., Willis-Owen C.A., Bendall S. (2015). Patients’ preferred mode of travel to the orthopaedic theatre. World J. Orthop..

[26] Kojima Y., Ina H., Fujita T., Mitono H. (2002). Relieving anxiety by entering the operating room on foot. Can. J. Anaesth..

[27] Biddiss E., Knibbe T.J., McPherson A. (2014). The effectiveness of interventions aimed at reducing anxiety in health care waiting spaces: A systematic review of randomized and nonrandomized trials. Anesth. Analg..

[28] Kaës R. (2007). Du Moi-peau aux enveloppes psychiques. Genèse et développement d’un concept. Le Carnet Psy.

[29] Ciccone A. (2001). Enveloppe psychique et fonction contenante: Modèles et pratiques. Cah. Psychol. Clin..

[30] Soltner C., Giquello J.A., Monrigal-Martin C., Beydon L. (2011). Continuous care and empathic anaesthesiologist attitude in the preoperative period: Impact on patient anxiety and satisfaction. Br. J. Anaesth..

